# Supplementary material for: Identification of photoperiod-induced specific miRNAs in the adrenal glands of Sunite sheep (Ovis aries)
Source: Front Vet Sci. 2022 Jul 22;9:888207. doi: 10.3389/fvets.2022.888207 (PMC9354845; doi:10.3389/fvets.2022.888207)
Supplement: Supplementary file 1 [file Data_Sheet_1.ZIP › Supplementary file/miRDeep2 file.zip!/miRDeep2 file/Know_Structure/SPLP42A3/oar-mir-758.pdf]

miRBase precursor : oar-mir-758
Total read count : 465
oar-miR-758-5p read count : 0
oar-miR-758-3p read count : 458
remaining reads : 7

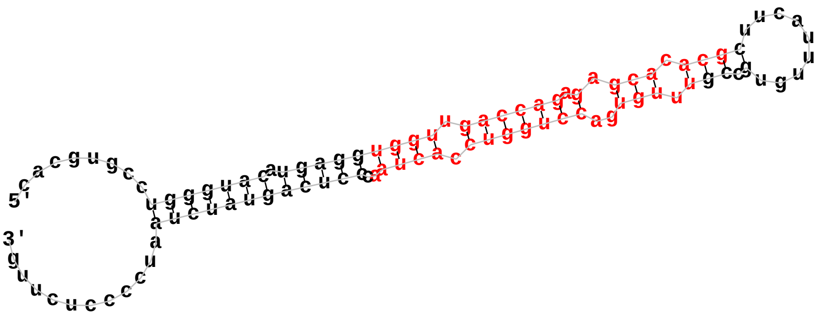

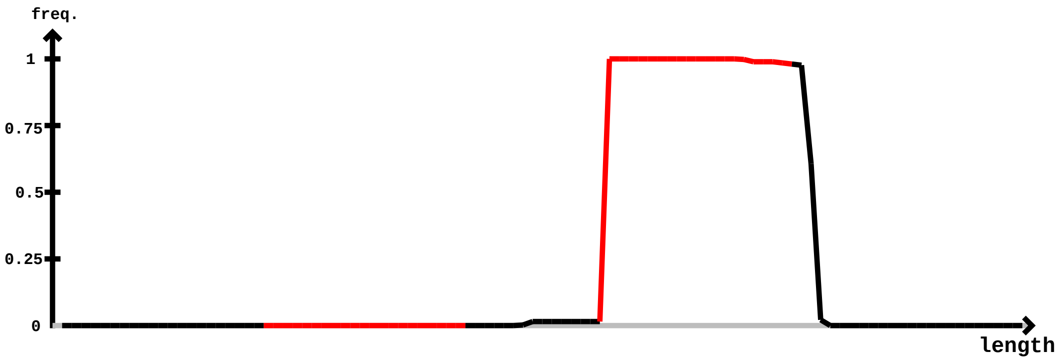

oar-miR-758-3p
oar-miR-758-5p

```
5'- cacgugccuggguacaugaggugguugaccagagagcacacgcuucauuugugccguuugugaccugguccacuaacccucaguaucuaauccccucuug   -3'    exp
    ........((((((.(((((((((.((((((.(.(((.((((.........).))).)))..))))))).))))..)))))))))))))...........    reads    mm    sample
    ...............................................uuugugccguuugugaccugguc..............................    1        0     seq
    ................................................uugugccguuugugaccuggucc.............................    4        0     seq
    ................................................uugugccguuugugaccugguccacu..........................    2        0     seq
    ........................................................uuugugaccugguccacua.........................    2        0     seq
    ........................................................uuugugaccugguccacuaa........................    2        0     seq
    ........................................................uuugugaccugguccacuaac.......................    172      0     seq
    ........................................................uuugugaccugguccacuaacc......................    272      0     seq
    ........................................................uuugugaccugguccacuaaccc.....................    10       0     seq
```
